# Psychiatric symptoms in vascular dementia. A new perspective

**DOI:** 10.1192/j.eurpsy.2025.1740

**Published:** 2025-08-26

**Authors:** C. M. Martín Gozalo, E. L. Bori, P. G. Melero, S. P. González, A. G. Ontaneda

**Affiliations:** 1 Psychiatry; 2 Geriatrics, Hospital Clínico San Carlos, Madrid, Spain

## Abstract

**Introduction:**

Psychiatric symptoms in vascular dementia occur in up to 95 % of patients. These symptoms can be of a depressive or manic type, among others. For this reason, it is essential to carry out a proper differential diagnosis between vascular dementia and other types of pathology that include psychiatric symptoms.

**Objectives:**

1. To describe the main psychiatric symptoms that could guide the diagnosis of vascular dementia.
2. To make an appropriate differential diagnosis in order to carry out the most suitable therapeutic approach in each specific case.

**Methods:**

A review of the most recent literature related to psychiatric symptomatology in patients with vascular dementia.

**Results:**

Vascular dementia can present with very diverse psychiatric pathology. Depending on the subcortical area affected, a particular symptomatology will predominate. For this reason, it is of vital importance to carry out a proper differential diagnosis. When the brain area affected is the ventromedial prefrontal cortex, the predominant symptomatology is depressive, with a higher percentage of patients with abulia. If the area most affected is the orbitofrontal cortex, disinhibition will predominate. However, if it is the dorsolateral prefrontal area, it will lead to executive dysfunctions.

On the other hand, it should be noted that psychiatric symptomatology due to vascular damage often has an atypical presentation in patients. For example, if what predominates is depressive symptomatology, what might appear relatively frequently would be late onset anxiety, irritability, or excessive somatic preoccupation. However, sadness or crying would not be as representative. If what predominates is the manifest symptomatology, in this case, with a high probability it would manifest itself in the form of behavioural disinhibition.

Because of these peculiarities, it is essential to make a proper screening between vascular dementia, late onset depression or Alzheimer’s disease, as the therapeutic approach to each pathology will be very different, as will be the prognosis.

**Conclusions:**

- Atypical psychiatric symptomatology may be the key to a diagnosis of vascular dementia.
- A proper differential diagnosis between vascular dementia, late onset depression and Alzheimer’s disease is essential.
- There is no clear benefit in the use of ACE inhibitors and NMDA receptor antagonists in cognitive impairment. However, there is evidence of improvement in cognitive function with SSRI antidepressants in patients with and without depression.

**Disclosure of Interest:**

None Declared

